# Low‐concentration atropine eyedrops for myopia control in a multi‐racial cohort of Australian children: A randomised clinical trial

**DOI:** 10.1111/ceo.14148

**Published:** 2022-09-09

**Authors:** Samantha Sze‐Yee Lee, Gareth Lingham, Magdalena Blaszkowska, Paul G. Sanfilippo, Adrian Koay, Maria Franchina, Audrey Chia, James Loughman, Daniel Ian Flitcroft, Christopher J. Hammond, Augusto Azuara‐Blanco, Julie M. Crewe, Antony Clark, David A. Mackey

**Affiliations:** ^1^ Centre for Ophthalmology and Visual Sciences(incorporating the Lions Eye Institute) University of Western Australia Perth Western Australia Australia; ^2^ Centre for Eye Research Ireland, School of Physics, Clinical and Optometric Sciences Technological University Dublin Dublin Ireland; ^3^ Centre for Eye Research Australia, University of Melbourne Royal Victorian Eye and Ear Hospital East Melbourne Victoria Australia; ^4^ Geraldton Eye Surgery Geraldton Western Australia Australia; ^5^ Singapore National Eye Centre Singapore Singapore; ^6^ Singapore Eye Research Institute Singapore Singapore; ^7^ Department of Ophthalmology Children's Health Ireland at Temple Street Dublin Ireland; ^8^ Departments of Ophthalmology and Twin Research and Genetic Epidemiology King's College London, St. Thomas' Hospital London UK; ^9^ School of Medicine Dentistry and Biomedical Science Queen's University Belfast Belfast UK; ^10^ School of Medicine, Menzies Research Institute Tasmania University of Tasmania Hobart Tasmania Australia

**Keywords:** atropine, axial length, myopia, myopia control, randomised controlled trial

## Abstract

**Background:**

To test the hypothesis that 0.01% atropine eyedrops are a safe and effective myopia‐control approach in Australian children.

**Methods:**

Children (6–16 years; 49% Europeans, 18% East Asian, 22% South Asian, and 12% other/mixed ancestry) with documented myopia progression were enrolled into this single‐centre randomised, parallel, double‐masked, placebo‐controlled trial and randomised to receive 0.01% atropine (*n* = 104) or placebo (*n* = 49) eyedrops (2:1 ratio) instilled nightly over 24 months (mean index age = 12.2 ± 2.5 and 11.2 ± 2.8 years, respectively). Outcome measures were the changes in spherical equivalent (SE) and axial length (AL) from baseline.

**Results:**

At 12 months, the mean SE and AL change from baseline were −0.31D (95% confidence interval [CI] = −0.39 to −0.22) and 0.16 mm (95%CI = 0.13–0.20) in the atropine group and −0.53D (95%CI = −0.66 to −0.40) and 0.25 mm (95%CI = 0.20–0.30) in the placebo group (group difference *p* ≤ 0.01). At 24 months, the mean SE and AL change from baseline was −0.64D (95%CI = −0.73 to −0.56) and 0.34 mm (95%CI = 0.30–0.37) in the atropine group, and −0.78D (95%CI = −0.91 to −0.65) and 0.38 mm (95%CI = 0.33–0.43) in the placebo group. Group difference at 24 months was not statistically significant (*p* = 0.10). At 24 months, the atropine group had reduced accommodative amplitude and pupillary light response compared to the placebo group.

**Conclusions:**

In Australian children, 0.01% atropine eyedrops were safe, well‐tolerated, and had a modest myopia‐control effect, although there was an apparent decrease in efficacy between 18 and 24 months, which is likely driven by a higher dropout rate in the placebo group.

## 1 INTRODUCTION

Randomised placebo‐controlled trials, including the Atropine for the Treatment of Myopia (ATOM) study in Singapore,^1^, ^2^, ^3^, ^4^, ^5^ Low‐concentration Atropine for Myopia Progression (LAMP) study in Hong Kong,^6^, ^7^ and the Indian‐ATOM study,^8^, ^9^ have supported the use of low‐concentration atropine eyedrops for myopia control. The LAMP study additionally compared 0.01% atropine to concentrations of 0.025% and 0.05%, and observed the latter had greatest myopia control benefit while maintaining relatively few adverse effects.^6^, ^7^ Thus, some clinicians concluded that 0.05% is the preferred atropine concentration for myopia control.^10^ However, this conclusion was based on studies conducted in Asia^1^, ^2^, ^3^, ^4^, ^5^, ^6^, ^7^, ^8^, ^9^ and may not be generalisable to other ancestries, as Asian children tend to spend less time outdoors^11^, ^12^ and because darker iris pigmentations bind more atropine, resulting in lower drug availability within the eye.^13^ To date, studies on atropine eyedrops for myopia control in children of non‐Asian ancestries were non‐randomised or did not include a placebo‐control group.^14^, ^15^, ^16^, ^17^, ^18^

The Western Australia (WA)‐ATOM study^11^ is a placebo‐controlled trial that aims to address this by testing the hypothesis that nightly instillation of 0.01% atropine eyedrops is a safe and effective myopia‐control therapy in a multi‐racial cohort of Australian children with myopia. This paper reports the results from the first 24 months of the trial.

## 2 METHODS

This single‐centre, double‐masked, randomised, placebo‐controlled trial had two parallel arms: 0.01% atropine eyedrops and placebo eyedrops. A conservative decision to only include the 0.01% concentrations was made to minimise the risks of adverse effects in our sample, given Australia's high levels of sunlight and outdoor lifestyle, and that 0.01% atropine eyedrops already have significant impact on pupil size and responsiveness in adults in Ireland after only 5 days of daily instillation.^19^ Both eyedrops contained the same vehicle and 0.01% benzalkonium chloride as a preservative. The placebo and treatment eyedrops were packaged identically, with only the participants' names and addresses labelled on the bottles. The detailed protocol and participants' baseline characteristics of the study have been published.^11^ A minimum sample of 103 participants was required based on the sample size calculation.^11^ We increased this to 150 participants to account for missing data and attrition. Children who were 6–16 years old at baseline, with spherical equivalent (SphE) ≤−1.50 D, astigmatism ≤1.50 DC and documented myopia progression ≥0.50 D year^−1^ were recruited. Participants who did not meet these criteria, who were unable to complete the eye tests, had ocular or systemic co‐morbidities (including amblyopia and strabismus), or had previously used atropine eyedrops or orthokeratology contact lenses were not eligible for enrolment. Cover test was performed prior to randomisation should the examiner suspect heterotropia, and children with manifest stabismus were not enrolled.

At enrolment, parents or caregivers of participants signed an informed consent after they were provided a full explanation of the nature of the study, while the participants provided verbal assent. This trial was approved by the University of Western Australia Human Research Ethics Committee, conducted in accordance to the tenets of the Declaration of Helsinki, and registered on the Australia and New Zealand Clinical Trials Registry (no. ACTRN12617000598381). Use of placebo and 0.01% atropine eyedrops were approved by the Therapeutics Goods Administration, Department of Health, Australia.

At enrolment, participants were allocated to the treatment or placebo group at a 2:1 ratio using a simple randomisation process.^11^ Participants' ancestry was classified into Europeans, East Asians, South Asians, and other/mixed ancestry,^11^ based on parent/caregiver‐reported information. These categories were chosen to allow comparison to previous similar studies conducted in East Asia (the ATOM study in Singapore^1^, ^2^, ^3^, ^4^ and LAMP study in Hong Kong^6^, ^7^, ^20^) and South Asia (India‐ATOM study^21^). The other/mixed group comprised participants of African, Arabians, Hispanics, Southeast Asian, and any combination of mixed European/Asian/Maori ancestries. Participants were seen again at 6, 12, 18 and 24 months. To increase compliance to their scheduled visits, participants were sent text message reminders and 1 day prior to their scheduled visits, and participants who missed their scheduled appointments were contacted by telephone and/or email to reschedule to a later time. This first 24 month is the treatment phase of the study, during which participants used the allocated eyedrops on a nightly basis in both eyes.

### 2.1 Eye examination

Distance and near visual acuities (VA; logMAR‐style charts) were measured monocularly at each visit using the participants' habitual distance correction and then with pinholes over their optical correction. The better of the two measurements (with habitual correction or habitual correction + pinholes) was taken as the best‐corrected VA (BCVA).^22^, ^23^ Accommodative amplitude (Royal Air Forces rule [Good‐Lite Elgin, Illinois]), pupillary measures (NPi‐200 digital pupillometer [NeurOptics Inc., Laguna Hills, California]), axial length (AL) and anterior chamber depth (ACD; IOLMaster V5 [Carl Zeiss Meditec AG, Jena, Germany]), and cycloplegic autorefraction (Nidek ARK‐510A, NIDEK Co. Ltd, Japan) were measured at each visit. Autorefraction was performed at least 20 min after the instillation of 1–3 drops of 1% cyclopentolate in each eye, with the number of eyedrops instilled dependent on the extent of cycloplegia achieved. This was assessed by the examiner using a pen torch to observe pupil responses. At the baseline and 24‐month visits, crystalline lens thickness and central corneal thickness were measured using Scheimflug imaging (Oculus Pentacam [software version 6.08r27; OculusOptikgerate GmbH, Wetzlar, Germany]) and stereoacuity was measured using a Titmus Fly Stereotest. Pupillary measures, include mesopic pupil size and pupil light reactions, were measured in a dark room. Participants were instructed to fixate at a small, distant (≥3 m) red target for 5 s, while a 50‐mW white light stimulus lasting 0.8‐s was used to trigger pupil light response, and the device automatically measured pupil size, and the latency and velocity of constriction. The onset of constriction was defined as a decrease of 5% of the initial baseline pupil size (confirmed by email from the manufacturer).

### 2.2 Quality‐of‐life questionnaire and adverse events

At each visit following randomisation, parents/caregivers completed the amblyopia treatment index (ATI) questionnaire. This is a standardised questionnaire used to assess the impact of patching or atropine treatment on children with amblyopia and their families.^24^ The ATI comprises 18 questions with a 5‐point likert scale response (strongly agree to strongly disagree) and includes questions on the child's acceptability and tolerance to eyedrops and impact on daily activities. Out of the 18 questions, two questions (‘other children stare at my child when the drops are in’ and ‘using the drops makes it difficult for my child to play with blocks or toys’) were not relevant to the study cohort and thus not analysed (Table S1). A free‐text comment box was available at the end of the questionnaire for parents/caregivers to list other ocular complaints or incidents.

### 2.3 Statistical analysis

The main efficacy outcome measures were the changes in SphE and AL. Safety and tolerability measures included changes in distance and near BCVA, accommodative amplitude, pupillary light reactions, and parent/caregiver‐reported incidents.

An intention‐to‐treat analysis was conducted using R (version 4.1.1; 2021 The R Foundation for Statistical Computing Platform [https://www.r-project.org/]), with level of significance set at *p* < 0.05. The amount of change in ocular measures from baseline was calculated at each visit and used as the outcome measure. The effect of treatment on myopia progression was analysed using linear mixed models, which account for the longitudinal (repeated) measurements. A random intercept term with nesting for eyes within individuals was included in the models to account for the clustered nature of the measurements from the eyes of the same person. Baseline measurement of the ocular outcome and any demographic variables that were significantly different at baseline were included in the models as fixed‐effect covariates.

## 3 RESULTS

Enrolled participants completed the baseline visit between June 2017 and December 2019. (See Figure S1 for participant numbers at each visit and reasons for participant exclusion or withdrawal in the footnotes.) Of the 153 enrolled participants, 104 (68.0%) and 49 (32.0%) were randomised to receive 0.01% atropine and placebo eyedrops, respectively. Participants in the placebo group were, on average, 1 year older and started wearing spectacles at an older age compared to those in the atropine group (Table 1). To account for this age difference between groups, baseline age was included as a fixed‐effect covariate in all models. Additionally, those in the placebo group had thinner crystalline lens, deeper anterior chambers, and longer constrition lat at baseline than those in the atropine group (Table 1). There was no other significant difference in demography or ocular measures at baseline between groups.

**TABLE 1:** Participant characteristic according to treatment randomisation

	Placebo (*n* = 49)	Atropine 0.01% (*n* = 104)	*p*‐value
Demography			
Index age (years)^a^	Mean = 12.2 (SD: 2.5)	Mean = 11.2 (SD: 2.7)	0.031*
Self‐reported age at first pair of spectacles (years)^a^	Mean = 8.76 (SD: 2.1)	Mean = 7.8 (SD: 2.4)	0.021*
Boys (*n*, %)^b^	20 (41.7%)	44 (42.3%)	1.00
Parental myopia (*n*, %)^b^ ^,^ ^c^			0.61
None	12 (24.5%)	18 (17.3%)	
1 parent	20 (40.8%)	44 (42.3%)	
Both parents	17 (34.7%)	40 (38.5%)	
Unknown	0 (0.0%)	2 (0.2%)	
Ethnicity (*n*, %)			0.99
European	23 (46.9%)	52 (50.0%)	
East Asian	9 (18.4%)	18 (17.3%)	
South Asian	11 (22.4%)	22 (21.1%)	
Other^d^	6 (12.2%)	12 (11.5%)	
Study completion^e^			
Completed 6‐month	45 (91.8%)	101 (97.1%)	0.21
Completed 12‐month	41 (83.7%)	97 (93.3%)	0.08
Completed 18‐month	40 (81.6%)	97 (93.3%)	0.047*
Completed 24‐month	37 (75.5%)	94 (90.4%)	0.024*
Ocular measures (median [IQR])^f^			
Myopia progression prior to enrolment (D year^−1^)	−1.00 (−0.75 to −1.29)	−0.91 (−0.67 to −1.30)	0.94
Spherical equivalent (D)	−3.56 (−4.56 to −2.75)	−3.13 (−4.08 to −2.48)	0.25
Axial length (mm)	24.7 (24.4–25.4)	24.6 (24.2–25.2)	0.19
Distance BCVA^g^ (logMAR)	0.02 (−0.01 to 0.06)	0.02 (−0.03 to 0.07)	0.75
Near BCVA^g^ (logMAR)	0.04 (0.00–0.01)	0.08 (0.04–0.13)	0.06
Accommodative amplitude (D)	16.7 (14.3–20.0)	14.8 (12.5–20.0)	0.07
Stereoacuity (arcmin)	40 (40–40)	40 (40–40)	0.24
Pupillary light response			
Constriction latency (s)	0.230 (0.215, 0.235)	0.215 (0.200, 0.230)	0.010*
Constriction velocity (mm s^−1^)	−3.08 (−3.59, −2.64)	−3.14 (−3.48, −2.79)	0.75
Dilation velocity (mm s^−1^)	1.31 (1.20, 1.43)	1.30 (1.11, 1.48)	0.36
Amplitude (mm)	2.35 (2.15, 2.73)	2.43 (2.20, 2.70)	0.72

*Note*: Statistically significant at *p < 0.05 or **p < 0.01.

Abbreviations: ACD, anterior chamber depth; BCVA, best‐corrected visual acuity; IQR, interquartile range; SD, standard deviation; VA, best‐corrected visual acuity.

^a^ Group difference analysed using independent *t*‐test.

^b^ Group difference analysed using chi‐square test.

^c^ Information on parental myopia collected using questionnaires and verified using autorefraction on parents; information on parental myopia not available for two participants as these children were adopted.

^d^ Includes those of African (*n* = 1), Arabian (*n* = 4), Hispanic (*n* = 1), Southeast Asian (*n* = 6) and any combination of mixed Caucasian/Asian/Moari (*n* = 6) ancestries.

^e^ Fisher exact test.

^f^ Group difference analysed using linear mixed effect models to account for within‐subject correlation between two eyes (except for accomodative amplitude and steoacuity analysed using Wilcox rank sum test).

^g^ BCVA estimated by measuring VA with pinholes over the presenting optic correction.

Over the 24 months, 22 participants withdrew from the study (Figure S1), including 10 (9.7%) in the atropine group and 12 (24.5%) in the placebo group. This differential rate of withdrawal became statistically significant at 18 and 24 months (*p* = 0.047 and 0.024, respectively; Table 1). There was no significant difference in age, sex, or ancestry between participants who completed the treatment phase (first 24 months) and those who withdrew (*p* ≥ 0.06). However, participants in placebo and atropine groups who completed the 24‐month treatment phasehad relatively stable myopia (progression in SphE by ~0.5 D over 24 months), compared to those who withdrew by 12 or 18 months (progression of ~1.0 D by 18 months; Figure S2).

There were also three participants who relocated interstate during the course of the study. One of these participants relocated and thus withdrew between 6 and 12 months (included in the withdrawal count); one relocated between 12 and 18 months but returned to the study site for their eye examination at 24 months (although missing their 18‐month appointment), and one relocated after 18 months and was seen interstate for their 24‐month follow‐up using testing equipment of the same models at a collaborating institute.

### 3.1 Changes in myopia and ocular biometry

**FIGURE 1:**
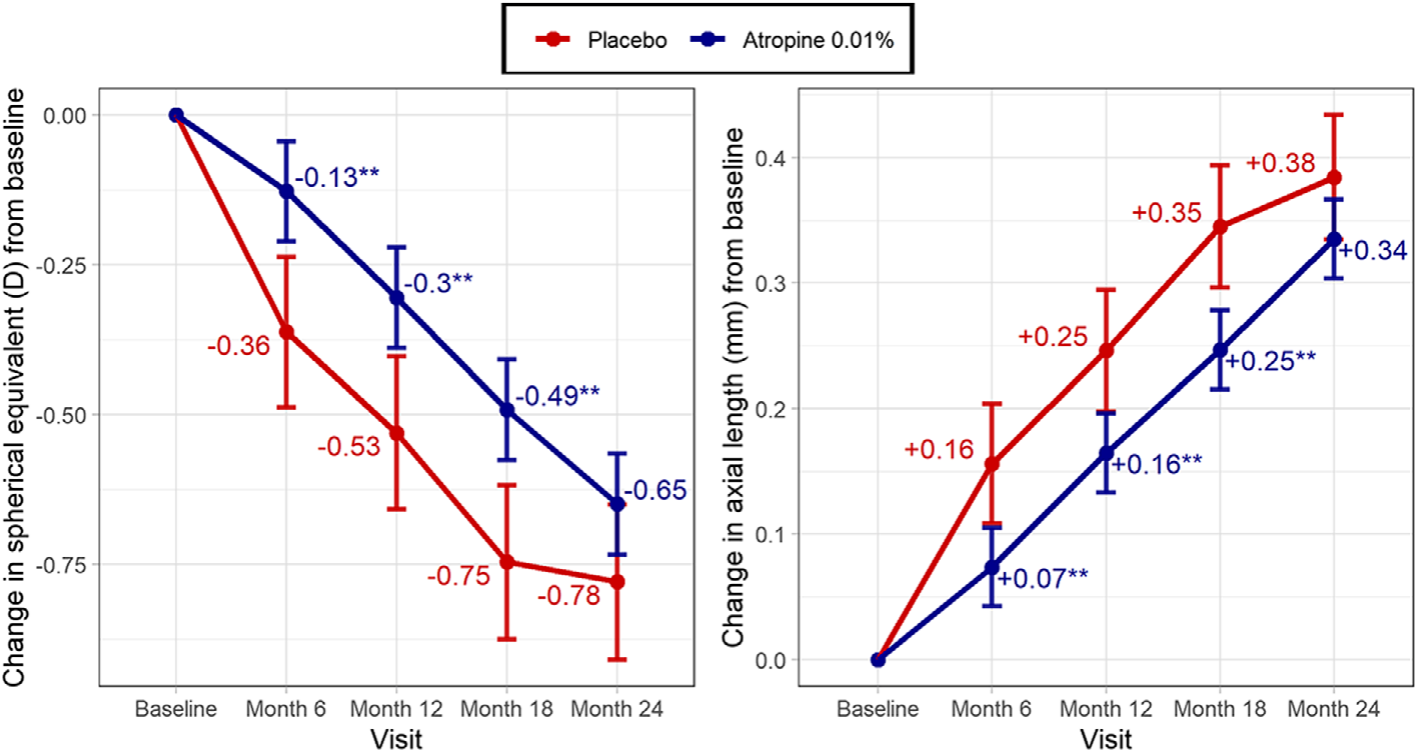
Estimated marginal mean change in spherical equivalent (left) and axial length (right) from baseline. Statistically different from the placebo group at *p < 0.05 or **p < 0.01. Estimates are adjusted for baseline value; error bars represent standard error

Over the study period, SphE decreased and AL increased significantly in both groups (*p* < 0.01; Figure 1 and Table 2). The atropine group had slower progression than the placebo group at all visits, but this difference failed to reach statistical significance at 24 months. This lack of significance at 24 months seemed to be driven by a slowing of progression in the placebo group in the last 6 months of the treatment period (Figure 1). There was no significant treatment × visit interaction on SphE or AL progression, suggesting that the main effect of atropine 0.01% treatment did not significantly increase or decrease over time. There was also no interaction effect between the treatment group and age or sex on change in SphE or AL (*p* ≥ 0.48).

**TABLE 2:** Cumulative change in spherical equivalent and axial length from baseline

Visit	Estimated marginal means		Difference^a^	*p*‐value^a^
	Placebo	Atropine 0.01%		
Spherical equivalent (D)^b^				
6 months	−0.36 (95%CI = −0.49 to −0.24)	−0.13 (95%CI = −0.21 to −0.04)	0.24 (95%CI = +0.08 to +0.40)	0.003*
12 months	−0.53 (95%CI = −0.66 to −0.40)	−0.31 (95%CI = −0.39 to −0.22)	0.23 (95%CI = +0.07 to +0.39)	0.004*
18 months	−0.74 (95%CI = −0.87 to −0.61)	−0.49 (95%CI = −0.58 to −0.41)	0.25 (95%CI = +0.10 to +0.42)	0.002*
24 months	−0.78 (95%CI = −0.91 to −0.65)	−0.64 (95%CI = −0.73 to −0.56)	0.14 (95%CI = −0.03 to +0.29)	0.09
Axial length (mm)^b^				
6 months	0.16 (95%CI = 0.11–0.20)	0.07 (95%CI = 0.04–0.11)	−0.08 (95%CI = −0.14 to −0.02)	0.005*
12 months	0.25 (95%CI = 0.20–0.30)	0.16 (95%CI = 0.13–0.20)	−0.08 (95%CI = −0.14 to −0.02)	0.006*
18 months	0.35 (95%CI = 0.30–0.39)	0.25 (95%CI = 0.22–0.28)	−0.10 (95%CI = −0.16 to −0.04)	0.001*
24 months	0.38 (95%CI = 0.33–0.43)	0.34 (95%CI = 0.30–0.37)	−0.05 (95%CI = −0.11 to +0.01)	0.1

*Note*: *p < 0.05 group difference.

Abbreviation: CI, confidence interval.

^a^ Group difference analysed using linear mixed effect models, corrected for age and baseline measures.

^b^ Change from baseline statistically significant at all visits.

We additionally conducted a subgroup analysis for children ≤10 years old at baseline, given the reports that atropine eyedrops may be more effective in young children. Similar outcomes were observed to those of the analyses involving the entire cohort, but with larger effect sizes (Figure S3).

The ACD similarly increased over the 24 months in both groups. However, the atropine group had a greater increase in ACD than the placebo group at 24 months (change from baseline: 0.045 vs. 0.012 mm, *p* = 0.018; Table 3). The cornea was significantly flatter by 0.008 mm at the end of the 24‐month treatment phase in both groups (Table 3), with no significant difference between groups. Lens thickness increased in both groups over the study period (placebo: 0.042 mm, atropine: 0.052 mm, both *p* ≤ 0.01), with no significant difference between groups. There was no significant change over time or group difference in central corneal thickness.

**TABLE 3:** Cumulative change in other ocular measures from baseline

Visit	Estimated marginal means (95%CI)		*p*‐value
	Placebo	0.01% atropine	
Ocular biometrics			
Anterior chamber depth (mm)			
6 months	0.025 (0.004–0.046)*	0.016 (0.002–0.030)*	0.50
12 months	0.020 (−0.002 to 0.041)	0.038 (0.023–0.052)**	0.18
18 months	0.025 (0.003–0.047)*	0.046 (0.031–0.060)**	0.13
24 months	0.026 (0.003–0.049)*	0.044 (0.030–0.059)**	**0.018**
Central corneal radius (mm)			
6 months	−0.004 (−0.011 to 0.004)	−0.004 (−0.009 to 0.001)	0.99
12 months	0.001 (−0.007 to 0.009)	−0.001 (−0.006 to 0.005)	0.76
18 months	0.000 (−0.008 to 0.008)	0.006 (0.001–0.011)*	0.22
24 months	0.008 (0.000 to −0.016)*	0.008 (0.003–0.013)**	0.94
Lens thickness (mm)^a^			
24 months	0.042 (0.013–0.070)**	0.052 (0.035–0.070)**	0.53
Central corneal thickness (μm)^a^			
24 months	0.110 (−3.060 to 3.280)	0.360 (−1.570 to 2.300)	0.90
Pupillary measures			
Constriction latency (ms)			
6 months	0.012 (0.003–0.021)**	0.018 (0.012–0.024)**	0.31
12 months	0.004 (−0.005 to 0.014)	0.016 (0.010–0.022)**	**0.047**
18 months	0.006 (−0.003 to 0.016)	0.018 (0.012–0.024)**	**0.034**
24 months	0.012 (0.003–0.022)*	0.016 (0.010–0.022)**	0.59
Constriction velocity (mm s^−1^)^b^			
6 months	0.090 (−0.056 to 0.24)	0.280 (0.180–0.38)**	**0.035**
12 months	−0.030 (−0.190 to 0.13)	0.330 (0.240–0.43)**	**<0.001**
18 months	−0.020 (−0.170 to 0.14)	0.260 (0.160–0.36)**	**0.003**
24 months	0.030 (−0.130 to 0.19)	0.220 (0.120–0.32)**	**0.044**
Dilation velocity (mm s^−1^)			
6 months	−0.003 (−0.079 to 0.074)	0.047 (−0.004 to 0.097)	0.29
12 months	0.072 (−0.012 to 0.16)	0.043 (−0.010 to 0.095)	0.56
18 months	0.050 (−0.031 to 0.13)	0.034 (−0.018 to 0.086)	0.75
24 months	−0.030 (−0.110 to 0.055)	0.032 (−0.020 to 0.084)	0.22
Amplitude (mm)^c^			
6 months	−0.11 (−0.23 to 0.01)	−0.39 (−0.47 to −0.31)**	**<0.001**
12 months	−0.047 (−0.17 to 0.08)	−0.45 (−0.53 to 0.37)*	**<0.001**
18 months	−0.07 (−0.20 to 0.06)	−0.42 (−0.50 to −0.35)**	**<0.001**
24 months	−0.19 (−0.31 to −0.06)**	−0.37 (−0.45 to −0.29)*	**0.015**
Other ocular measures			
Distance BCVA (logMAR)			
6 months	0.01 (−0.01 to 0.02)	−0.02 (−0.04 to −0.01)**	**0.010**
12 months	−0.02 (−0.04 to 0.00)	−0.04 (−0.05 to −0.02)**	0.13
18 months	−0.02 (−0.04 to −0.01)*	−0.04 (−0.05 to −0.03)**	0.21
24 months	−0.03 (−0.05 to −0.01)**	−0.04 (−0.05 to −0.03)**	0.52
Near BCVA (logMAR)			
6 months	−0.03 (−0.05 to −0.01)**	−0.03 (−0.04 to −0.02)**	0.85
12 months	−0.04 (−0.06 to −0.02)*	−0.03 (−0.04 to −0.02)**	0.37
18 months	−0.01 (−0.04 to 0.01)	−0.01 (−0.03 to 0.00)	0.84
24 months	−0.03 (−0.06 to −0.01)	−0.03 (−0.04 to −0.02)**	0.65
Accommodative amplitude (D)			
6 months	−0.77 (−1.74 to 0.20)	−2.04 (−2.67 to −1.41)**	**0.033**
12 months	−0.18 (−1.18 to 0.82)	−2.04 (−2.68 to −1.39)**	**0.003**
18 months	−0.53 (−1.54 to 0.48)	−2.56 (−3.20 to −1.91)**	**0.001**
24 months	−1.38 (−2.41 to −0.34)**	−2.70 (−3.34 to −2.05)**	**0.039**
Stereoacuity (arcmin)^a^			
24 months	5.9 (−13.4 to 25.2)	13.7 (1.9–25.5)*	0.50

*Note*: Asterisks indicate statistical difference from baseline at *p < 0.05 and **p < 0.01. *p*‐values in bold represent significant group difference at *p* < 0.05. All analyses corrected for age and baseline measures.

Abbreviation: BCVA, best‐corrected visual acuity; CI, confidence interval.

^a^ Only measured at baseline and 24 months.

^b^ More negative values representing faster constriction velocity.

^c^ Difference between maximum and minimum pupil diameter.

### 3.2 Interaction with ancestry

A significant treatment × ancestry interaction effect on change in SphE and AL was noted (Figures S4 and S5). Participants of East Asian or South Asian descent showed no difference in change in spherical equivalent or axial length between the placebo and atropine groups throughout the 24 months (*p* > 0.05). In European participants, 0.01% atropine eyedrops significantly slowed progression up to 18 months, but not at 24 months. In participants of other/mixed ancestries, similar to the main effect as described above, there appeared to be a slight slowing in myopia progression in the placebo group in the final 6 months, which rendered a smaller difference between groups at 24 months.

### 3.3 Pupil size and light reaction

At all visits between 6 and 24 months, participants in the atropine group had significantly increased latency in pupillary constriction by ~0.17 s and decreased constriction velocity by 0.2–0.3 mm s^−1^ relative to baseline (*p* < 0.001 at all visits; Table 3). Amplitude of pupil diameter (difference between pupil size prior to light stimulus and pupil size at maximum constriction after light stimulus) was also significantly reduced by ~0.4 mm in the atropine group at all visits relative to baseline (*p* < 0.001–0.049; Table 3).

In the placebo group, there was a 0.12‐s increase in constriction latency relative to baseline at 6 and 24 months (*p* = 0.009 and 0.013; Table 3), but constriction velocity did not change significantly from baseline at any visit. Compared to baseline values, amplitude of pupil diameter was significantly reduced at 24 months by 0.2 mm in the placebo group (*p* = 0.003), but the difference in other visits were not significant. These changes in pupillary light response were greater in the atropine group than the placebo at most visits (Table 3). Dilation velocity did not change significantly from baseline in both groups nor was there was any significant difference in change in dilation velocity from baseline between groups (*p* > 0.05). There was no interaction effect between treatment and ancestry or eye colour on pupillary measures.

### 3.4 Other ocular effects of atropine 0.01% eyedrops

Both groups had small (1‐ to 2‐letter) improvements in distance and near BCVA throughout the study; however, there was no significant group difference (Table 3). Accommodative amplitude declined in both groups, although the atropine group had a greater reduction from baseline at each visit compared to the placebo group (Table 3). Between 6 and 24 months, the atropine group had a 2.0–2.7D decrease in accommodative amplitude from baseline, while the placebo group had little change in accommodative amplitude over the first 18 months (0.2–0.8D; *p* > 0.5), but this later decreased to 1.4D at 24 months compared to baseline (although not statistically significant). There was no interaction effect between treatment and ancestry or eye colour on accommodative measures.

### 3.5 Eyedrop tolerability and adverse events

There was no significant difference in responses to each ATI questionnaire item between groups. Based on these responses, both the placebo and atropine 0.01% eyedrops were well‐tolerated, with >90% of parents/caregivers in both groups reporting that their child did not seem to mind the eyedrops and that the eyedrops did not affect their child's activities, including near work, outdoor activities and learning (Table S1 shows responses at 24 months). There were nine adverse events in the treatment group reported during the study period, none of which were considered severe. Adverse events in the treatment group that were considered to be probably related to the study medication included two instances of a sore or heavy‐feeling eye and one report of blurred near vision. Other events in the treatment group were determined to be unlikely related to study medication, including two instances of allergic conjunctivitis (related to swimming) and one report each of a migraine, an asthma attack, appearance of visual floaters, and adnexal foreign body. In the placebo group, there was only one report of headaches, which was not deemed to be related to the study eyedrops. No other adverse events were reported in the placebo group, and there was no statistically significant difference in incident of adverse events between groups (treatment: 8.7% vs. placebo: 2.1%; group difference *p* = 0.17).

## 4 DISCUSSION

Previous randomised placebo‐controlled trials found that 0.01% atropine eyedrops slowed myopia progression in East or South Asian children, but had little impact on AL^1^, ^4^, ^6^, ^21^ (see Table 4 for comparison between the LAMP, ATOM, India‐ATOM, and current studies). In our multi‐racial cohort (~50% European), we found only a modest myopia control effect of 0.01% atropine which was not significant at 24 months. The lack of difference at 24 months could be due to several reasons. First, participants with faster myopia progression, who were more likely receiving the placebo, may have been more inclined to withdraw introducing attrition bias. Second, the placebo group were, on average, a year older than the treatment group, and, despite adjusting for baseline age, an age‐related slowing of myopia progression in the placebo group may have occurred in the later stages of the study. This notion is supported by our subgroup analysis, which shows that younger children had a slightly greater response to 0.01% atropine treatment compared to the whole cohort. Third, myopia treatment could become less effective over time. As summarised by Brennan et al.,^25^ most myopia‐control studies found the greatest effects in the first year of treatment. The authors^25^ posit that this could be due to an initial halt in eye growth when myopia treatment is commenced, but a continued relative shrinkage of the eye cannot be sustained for long‐term periods.

**TABLE 4:** Comparison of 0.01% atropine findings for myopia control compared to a placebo in different studies

Study	Cohort ancestry (*n*)^a^	Effect of 0.01% atropine versus placebo (difference in average annual change)	
		SphE (D)	AL (mm)
Current study (Australia)	All participants (*n* = 153)	0.23*	−0.08*
	European (*n* = 75)	0.20	−0.10*
	Other/mixed (*n* = 18)	0.54*	−0.21*
	East Asians (*n* = 27)	0.09	0.03
	South Asians (*n* = 33)	0.09	0.02
ATOM2 (Singapore)^b^	Mostly East Asians (*n* = 284)	0.17*	−0.05
LAMP (Hong Kong)	East Asians (*n* = 190)	0.19*	−0.06
I‐ATOM (India)	South Asians (*n* = 92)	0.22*	−0.05

Abbreviations: AL, axial length; ATOM, atropine for the treatment of myopia study; I‐ATOM, India ATOM; LAMP, low‐concentration atropine for myopia progression study; SphE, spherical equivalent.

^a^ Only randomised placebo‐controlled trials are shown; *n* refers to the total number of participants in the 0.01% atropine and placebo groups only.

^b^ 0.01% atropine and placebo trials did not run in parallel and axial lengths were measured using different instruments in atropine and placebo groups.

We also failed to find any significant treatment effect in children of East or South Asian ancestry. As our study was not powered to detect the difference between racial groups, the lack of a significant effect of treatment in our cohort of Asian children could be due to a small sample size, with fewer than 30 participants in each group. However, not only was there no statistical difference between treatment groups, the magnitude of difference in SphE between the placebo and control groups in our study was only about half that found in the other studies (0.09D vs. 0.17–0.22 D; Table 4). It is possible that low‐concentration atropine eyedrops do not have as great an effect on myopia progression in Asian children living in Australia relative to those living in Asia. This issue merits further investigation.

Previous meta‐analyses^26^, ^27^ concluded that atropine eyedrops were more effective in slowing down myopia progression in Asian children than in European children. However, the validity of those meta‐analyses may have been reduced by the fact that only two studies were conducted in European children and neither had placebo‐control groups. These studies also used 1% atropine, which is less well tolerated by children. Our findings suggest that participants of European or other/mixed ancestries derived greater benefits of 0.01% atropine eyedrops than East or South Asian children in terms of slowing down myopia progression and axial elongation, although our study was not powered to explore this. Given our small sample size in each ancestry group, further investigations are warranted to ascertain these finding and explore for possible explanations to these differences between ancestry.

The ACD significantly increased in both groups over the 2 years. This is in contrast to the data from LAMP and ATOM1 studies, which reported that the ACD decreased in individuals using atropine of various concentrations, although the changes were not statistically significant.^28^, ^29^ The effect of atropine on ACD is likely dependent on a complex interplay between the axial length growth and crystalline lens size and position. Deepening of the anterior chamber is associated with axial elongation; thus, we may expect the ACD to increase in both groups together with the axial length. However, the atropine group had a larger ACD increase, despite slower axial elongation in that group than the placebo group. This is likely because cycloplegic eyedrops, including atropine, cause the crystalline lens to move posteriorly as the ciliary muscle relaxes. Similar deepening effects on the anterior chamber have been reported following the instillation of cyclopentolate in children^30^ and atropine eyedrops for controlling post‐trabeculectomy ocular inflammation in adults.^31^

Most of the expected ocular effects of atropine eyedrops were observed, including reduced pupillary constriction and accommodative amplitude. However, these effects were small and not associated with complaints of photophobia or blurring of near vision. Moreover, near BCVA was significantly better in the atropine group at most study visits relative to baseline, and no difference in change in near BCVA or stereoacuity was noted between groups.

A strength of the study was the inclusion of children of various ancestry, which is reflective of the increasingly diverse Australian population.^32^ However, because the study was not powered to investigate the difference in treatment efficacy between ancestria groups, we had a relatively small sample of participants in each group. Further studies with larger samples of multi‐racial participants are warranted to confirm our findings.

A caveat of this study is the inclusion of a large participant age range (6–16 years at baseline). Given that the reported age of myopia stabilisation is around 16 years old,^33^ we may expect the older participants to have a natural slowing of myopia progression without any intervention. This age range was chosen to maximise participant recruitment rate and to include children in their later years of high school who may be facing more intense academic pressure and thus still subjected to myopia progression. Our analysis was further limited by a significant difference in baseline age between the placebo and treatment groups. While we have included baseline age in the statistical models, it might not fully account for the effect of age difference between groups. Additionally, there was a higher rate of participant withdrawal from the study in the placebo group. In Australia, prescribing low‐concentration atropine for myopia control is, anecdotally, already a widespread practice among optometrists and ophthalmologists, despite the lack of evidence in this population. Although parents/caregivers were unaware if their child was receiving the placebo or treatment eyedrops, some may have withdrawn to seek atropine eyedrops or other myopia interventions from their own eyecare practitioners after noting rapid progression of their child's myopia. Thus, the true myopia progression in the placebo group and, consequently, the benefits of 0.01% atropine, may have been underestimated in the current study. Future placebo‐control myopia treatment trials in countries like Australia, where use of low‐concentration atropine eyedrops for myopia control is widespread, may face a similar challenge of differential rates of withdrawal.

We did not explore for concentration‐dependent effects, as the LAMP study did,^6^, ^7^ which may inform on the value of varying atropine concentration in children with different risk levels. A conservative decision to not use higher concentrations in our study was made to minimise the risks of adverse effects in our sample, given Australia's high levels of sunlight and outdoor lifestyle, and that 0.01% atropine eyedrops already have significant impact on pupil size and responsiveness in European adults in Ireland after only 5 days of daily instillation.^19^ However, we noted that the medication was well‐tolerated by Australian children, alleviating concerns that low‐concentration atropine may cause more significant side effects in these children. Future studies could explore whether slightly higher concentrations of atropine, for example, 0.02%, may still be tolerated in Australian or European children while having greater efficacy.

We also did not assess adherence to eyedrop use. Given that atropine eyedrops has up to 2 weeks' duration of action, it is possible that missed therapy for a short duration for a few days, even when using low‐concentration atropine, may not impact treatment efficacy. Moreover, this does not affect the study's analytical plan given our intention‐to‐treat principle of analysis. Nonetheless, the effect of treatment adherence on efficacy is worth exploring in future studies, especially when comparing different concentrations of atropine.

### 4.1 Future directions and clinical relevance

Since the ATOM studies in Singapore,^1^, ^4^ 0.01% atropine eyedrops had been a preferred myopia treatment method, although the LAMP study showed that a slightly higher concentration of atropine (0.05%) had greater myopia control efficacy while still maintaining low rates of adverse effects.^6^, ^7^ The LAMP study also reported that 0.01% atropine had statistically significant but small myopia control benefits in East Asian children living in Hong Kong, with a slowing of spherical equivalent progression but not axial elongation relative to using a placebo. Almost identical findings for 0.01% atropine was found in children in India.^21^

The current study found that 0.01% atropine eyedrops had a modest myopia control effect in multi‐racial Australian children. The effect was greater at 12 and 18 months but reduced at 24 months. Our analysis showed that 0.01% atropine eyedrops were not effective for myopia control in South or East Asian children, and had only modest effects in European children and those of other/mixed ancestries. The LAMP study suggested that 0.05% atropine concentration may be effective in East Asian children in Hong Kong. It is unclear if this can be generalised to East Asian children living in Australia. Given the difference in magnitude of effect of 0.01% atropine eyedrops found in the current analysis versus the ATOM and LAMP studies, it remains possible that 0.05% atropine eyedrops could have a small or limited effect in East Asian children living in Australia. Addressing this important gap in our knowledge will help improve myopia control in these high‐risk children and enhance our appreciation of the anti‐myopigenic mechanism of atropine eyedrops.

Several randomised controlled trials on myopia control, including the current study, have consistently shown a relative benefit of 0.01% atropine over a placebo, even if the treatment effects are only moderate. Thus, it may now be scientifically and ethically sound for future trials to conduct non‐inferiority myopia control trials, rather than using placebo or single‐vision lenses as controls.

A next step for the current study is to monitor the current cohort of participants for potential rebound effects, which is anticipated given the findings from Phase 3 of the LAMP study, which showed that continued treatment in Asian children is preferred over a washout regime.^20^ Additionally, results from the current study will be combined with those from similar studies in Ireland^34^ and the United Kingdom,^35^ allowing a large and prospective independent participant meta‐analysis of low‐concentration atropine eyedrop efficacy for myopia control in Western populations.

## FUNDING INFORMATION

This study was funded by a Telethon‐Perth Children's Hospital Research Fund; the University of Western Australia's Faculty of Health and Medical Sciences (Early Career Researcher Small Grant Award); Healy Medical Research Foundation (Healy Research Collaboration Award); Australian Vision Research (previously the Ophthalmic Research Institute of Australia); the University of Western Australia (Research Collaboration Award). The funding organisations had no role in the design or conduct of this research. Dr. David A. Mackey is supported by a National Health and Medical Research Council (NHMRC, Australia) Practitioner Fellowship.

## CONFLICT OF INTEREST

The authors declare no potential conflict of interest.

## Supporting information


**Figure S1.** Participant numbers at each visit and number of withdrawals between each visit.
^a^Reasons for exclusion: 62 were ineligible at referral screening; 96 declined participation; and one was ineligible at baseline visit.
^b^Reasons for withdrawal prior to 6 months: one did not want diagnostic or study drops instilled; three had difficulty adhering to the treatment regimen; one had difficulty attending appointments + fear of receiving placebo; two were uncontactable.
^c^Reasons for withdrawal between 6 and 12 months: one relocated; two wanted to seek myopia treatment (atropine or orthokeratology) privately; one did not want diagnostic or study drops instilled; one had difficulty adhering to the treatment regimen; one had difficulty attending appointments; one was uncontactable; one did not provide reason
^d^Reasons for withdrawal between 12 and 18 months: one wanted to seek atropine eyedrops privately due to rapid myopia progression
^e^Reasons for withdrawal between 18 and 24 months: one did not want diagnostic or study drops instilled; one had difficulty attending appointments; two cited personal reasons; one was uncontactable; one did not provide reason

**Figure S2.** Mean change in spherical equivalent (top) and axial length (bottom) from baseline to last visit prior to withdrawal compared to those who completed 24 months. Error bars are ± 1 standard error. Note that seven participants (four placebo, three atropine) withdrew before 6 months for whom no inference can be made about their myopia progression.

**Figure S3.** Estimated marginal mean change in spherical equivalen (left) and axial length (right) from baseline for children 10 years or younger. Statistically different from the placebo group at *p< 0.05 or **p< 0.01. Estimates are adjusted for baseline value; error bars represent standard error.

**Figure S4.** Change in spherical equivalent (D) from baseline in participants of different ancestries, adjusted for age and spherical equivalent at baseline. Numbers indicate estimated marginal means; statistically different from the placebo group at *p < 0.05 or **p < 0.01. Estimates are adjusted for baseline value; error bars representing standard error

**Figure S5.** Change in axial length (mm) from baseline in participants of different ancestries, adjusted for age and axial length at baseline. Numbers indicate estimated marginal means; statistically different from the placebo group at *p < 0.05 or *p* < 0.01. Estimates are adjusted for baseline value; error bars represent standard error

**Table S1.** Parent‐ or guardian‐administered quality of life responses at 24 months. Note that total percentage may not add up to 100% due to ‘not applicable’ or ‘unsure’ responses. Note that there was no significant difference in any item response between the atropine and placebo groups.
