# Supplementary material for: Low‐concentration atropine eyedrops for myopia control in a multi‐racial cohort of Australian children: A randomised clinical trial
Source: Clin Exp Ophthalmol. 2022 Sep 9;50(9):1001–12. doi: 10.1111/ceo.14148 (PMC10086806; doi:10.1111/ceo.14148)
Supplement: Supplementary file 1 — Figure S1. Participant numbers at each visit and number of withdrawals between each visit. aReasons for exclusion: 62 were ineligible at referral screening; 96 declined participation; and one was ineligible at baseline visit. bReasons for withdrawal prior to 6 months: one did not want diagnostic or study drops instilled; three had difficulty adhering to the treatment regimen; one had difficulty attending appointments + fear of receiving placebo; two were uncontactable. cReasons for withdrawal between 6 and 12 months: one relocated; two wanted to seek myopia treatment (atropine or orthokeratology) privately; one did not want diagnostic or study drops instilled; one had difficulty adhering to the treatment regimen; one had difficulty attending appointments; one was uncontactable; one did not provide reason dReasons for withdrawal between 12 and 18 months: one wanted to seek atropine eyedrops privately due to rapid myopia progression eReasons for withdrawal between 18 and 24 months: one did not want diagnostic or study drops instilled; one had difficulty attending appointments; two cited personal reasons; one was uncontactable; one did not provide reason [file CEO-50-1001-s004.docx]

******

***Supplementary Figure 1. Participant numbers at each visit and number of withdrawals between each visit****.*

*^a^Reasons for exclusion: 62 were ineligible at referral screening; 96 declined participation; and 1 was ineligible at baseline visit*

*^b^Reasons for withdrawal prior to 6 months: 1 did not want diagnostic or study drops instilled; 3 had difficulty adhering to treatment regimen; 1 had difficulty attending appointments + fear of receiving placebo; 2 were uncontactable*

*^c^Reasons for withdrawal between 6 and 12 months: 1 relocated; 2 wanted to seek myopia treatment (atropine or orthokeratology) privately; 1 did not want diagnostic or study drops instilled; 1 had difficulty adhering to treatment regimen; 1 had difficulty attending appointments; 1 was uncontactable; 1 did not provide reason*

*^d^Reasons for withdrawal between 12 and 18 months: 1 wanted to seek atropine eyedrops privately due to rapid myopia progression*

*^e^Reasons for withdrawal between 18 and 24 months: 1 did not want diagnostic or study drops instilled; 1 had difficulty attending appointments; 2 cited personal reasons; 1 was uncontactable; 1 did not provide reason*
