# Supplementary material for: Low‐concentration atropine eyedrops for myopia control in a multi‐racial cohort of Australian children: A randomised clinical trial
Source: Clin Exp Ophthalmol. 2022 Sep 9;50(9):1001–12. doi: 10.1111/ceo.14148 (PMC10086806; doi:10.1111/ceo.14148)
Supplement: Supplementary file 2 — Figure S2. Mean change in spherical equivalent (top) and axial length (bottom) from baseline to last visit prior to withdrawal compared to those who completed 24 months. Error bars are ± 1 standard error. Note that seven participants (four placebo, three atropine) withdrew before 6 months for whom no inference can be made about their myopia progression. [file CEO-50-1001-s001.docx]

***
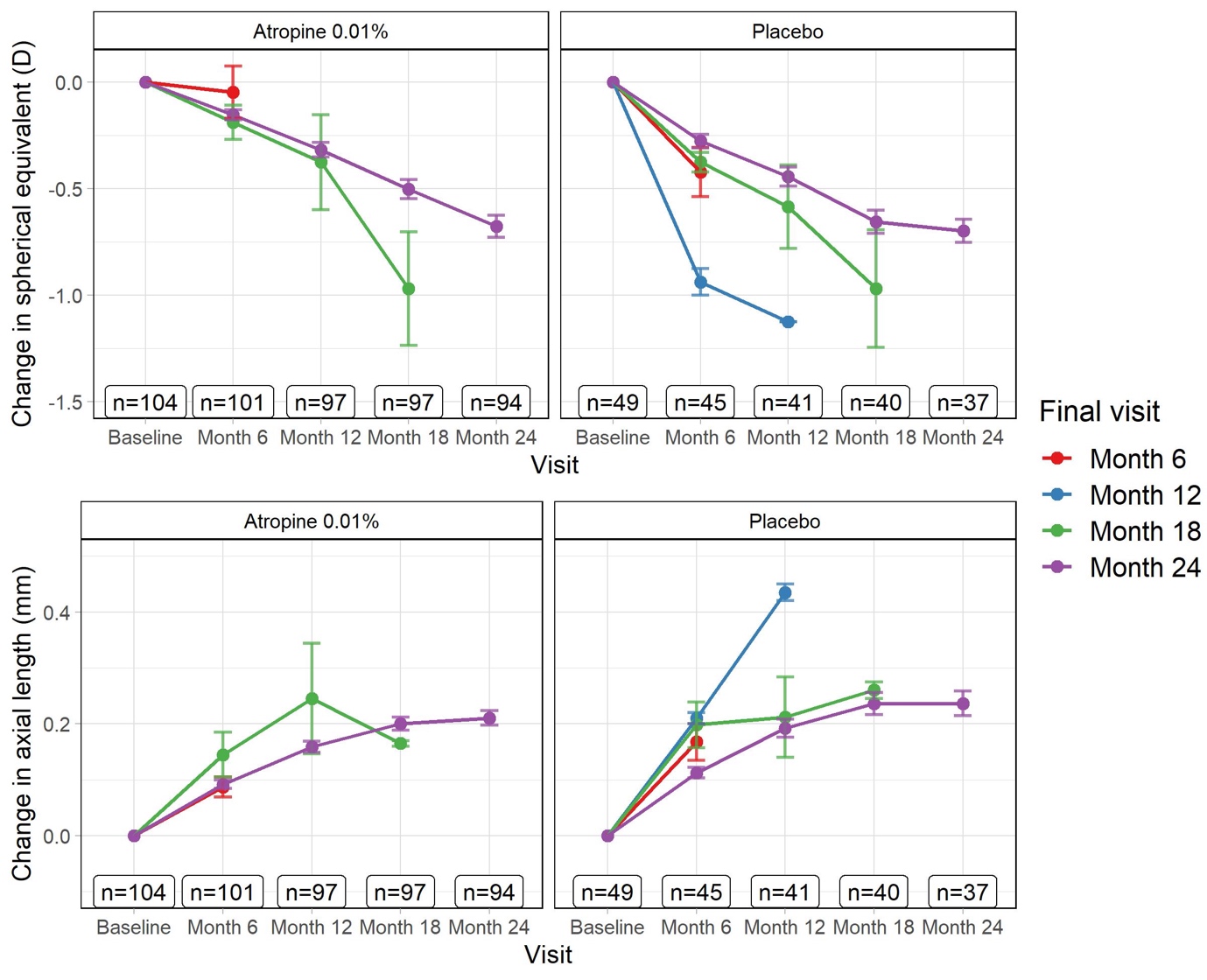
***

**Supplementary Figure 2**. Mean change in spherical equivalent (top) and axial length (bottom) from baseline to last visit prior to withdrawal compared to those who completed 24 months. Error bars are ± 1 standard error. Note that 7 participants (4 placebo, 3 atropine) withdrew before 6 months for whom no inference can be made about their myopia progression.
