# Supplementary material for: Low‐concentration atropine eyedrops for myopia control in a multi‐racial cohort of Australian children: A randomised clinical trial
Source: Clin Exp Ophthalmol. 2022 Sep 9;50(9):1001–12. doi: 10.1111/ceo.14148 (PMC10086806; doi:10.1111/ceo.14148)
Supplement: Supplementary file 3 — Figure S3. Estimated marginal mean change in spherical equivalen (left) and axial length (right) from baseline for children 10 years or younger. Statistically different from the placebo group at *p< 0.05 or **p< 0.01. Estimates are adjusted for baseline value; error bars represent standard error. [file CEO-50-1001-s002.docx]

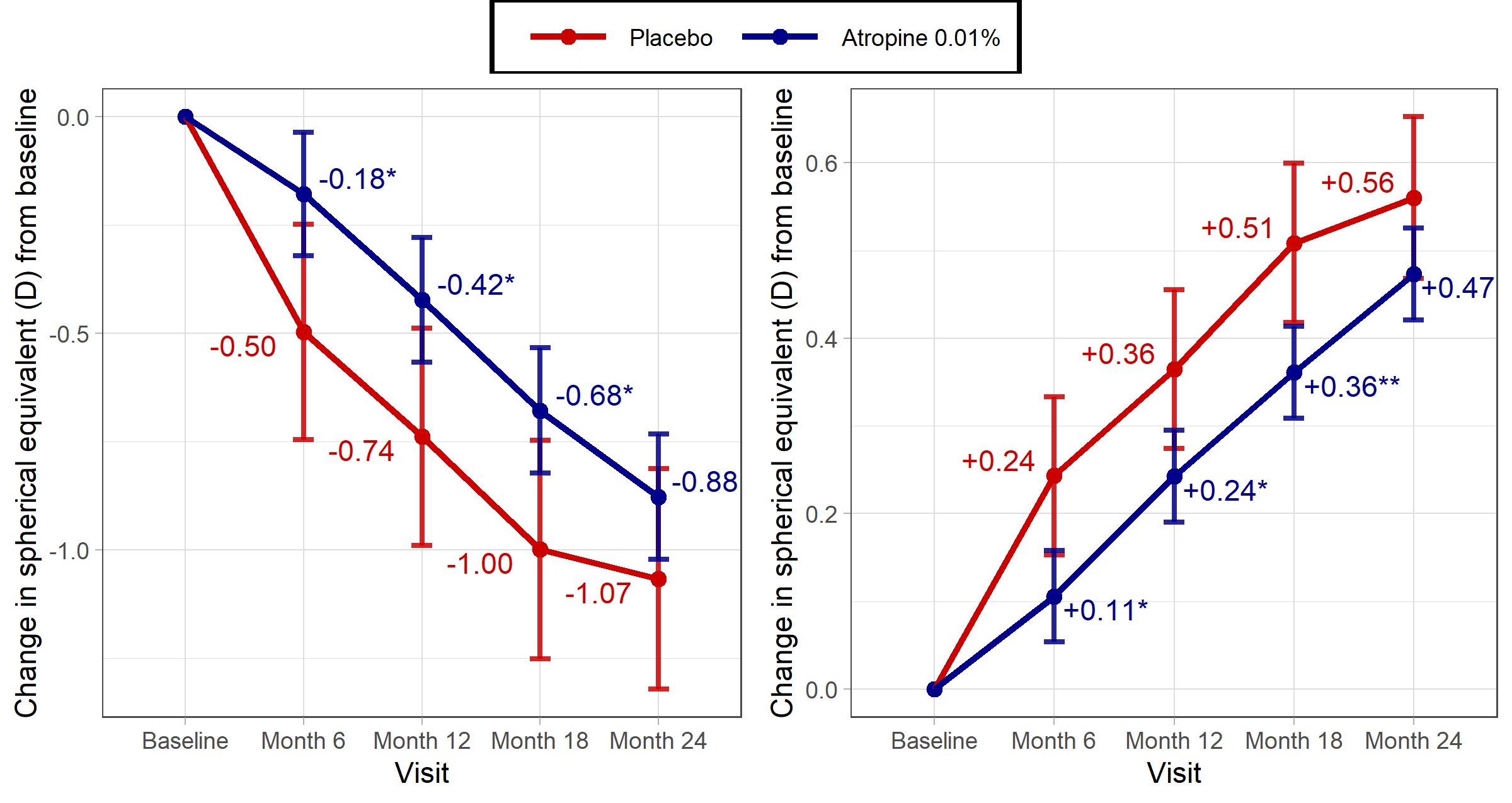


***Supplementary Figure 3****. Estimated marginal mean change in spherical equivalent (left) and axial length (right) from baseline for children 10 years old or younger. Numbers indicate estimated marginal means; statistically different from the placebo group at *p< 0.05 or **p< 0.01. Estimates are adjusted for baseline value; error bars representing standard error*
