# Supplementary material for: Low‐concentration atropine eyedrops for myopia control in a multi‐racial cohort of Australian children: A randomised clinical trial
Source: Clin Exp Ophthalmol. 2022 Sep 9;50(9):1001–12. doi: 10.1111/ceo.14148 (PMC10086806; doi:10.1111/ceo.14148)
Supplement: Supplementary file 5 — Figure S5. Change in axial length (mm) from baseline in participants of different ancestries, adjusted for age and axial length at baseline. Numbers indicate estimated marginal means; statistically different from the placebo group at *p < 0.05 or p < 0.01. Estimates are adjusted for baseline value; error bars represent standard error [file CEO-50-1001-s003.docx]

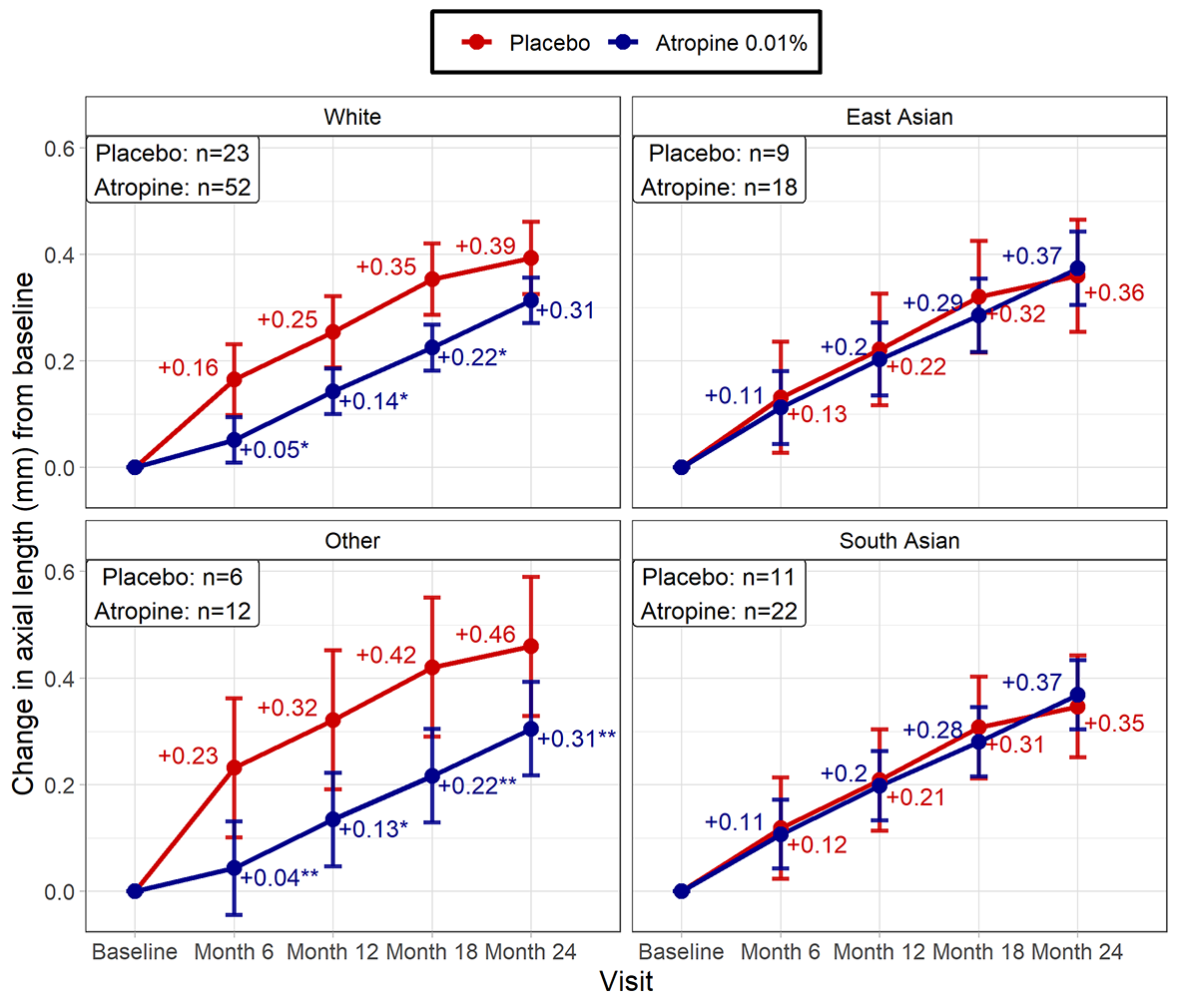


***Supplementary Figure 5****. Change in axial length (mm) from baseline in participants of different ancestries, adjusted for age and axial length at baseline.* *Numbers indicate estimated marginal means; statistically different from the placebo group at *p< 0.05 or p< 0.01. Estimates are adjusted for baseline value; error bars representing standard error*
