# Supplementary material for: Low‐concentration atropine eyedrops for myopia control in a multi‐racial cohort of Australian children: A randomised clinical trial
Source: Clin Exp Ophthalmol. 2022 Sep 9;50(9):1001–12. doi: 10.1111/ceo.14148 (PMC10086806; doi:10.1111/ceo.14148)
Supplement: Supplementary file 6 — Table S1. Parent‐ or guardian‐administered quality of life responses at 24 months. Note that total percentage may not add up to 100% due to ‘not applicable’ or ‘unsure’ responses. Note that there was no significant difference in any item response between the atropine and placebo groups. [file CEO-50-1001-s005.docx]

**Supplementary Table 1.** Parent- or guardian-administered quality of life responses at 24 months. Note that total percentage may not add up to 100% due to “not applicable” or “unsure” responses. Note that there was no significant difference in any item response between the atropine and placebo groups.

| **Item** | **Agree/ strongly agree** | **Neither agree/ disagree** | **Disagree/ strongly disagree** |
| --- | --- | --- | --- |
| 1. Child does not mind drops  - Placebo - Atropine 0.01% | 32 (94.1%)  72 (91.1%) | 1 (2.9%)  2 (2.5%) | 1 (2.9%)  5 (6.4%) |
| 1. Worry that child may miss out on fun activities  - Placebo - Atropine 0.01% | 0 (0.0%)  0 (0.0%) | 0 (0.0%)  0 (0.0%) | 33 (97.1%)  77 (97.5%) |
| 1. Drops affect child’s learning  - Placebo - Atropine 0.01% | 0 (0.0%)  0 (0.0%) | 0 (0.0%)  3 (3.8%) | 32 (94.1%)  75 (93.8%) |
| 1. Drops makes it hard to play outside  - Placebo - Atropine 0.01% | 0 (0.0%)  0 (0.0%) | 0 (0.0%)  2 (2.5%) | 31 (91.2%)  76 (95.0%) |
| 1. Trouble putting drops in child’s eyes  - Placebo - Atropine 0.01% | 2 (5.9%)  0 (0.0%) | 1 (3.0%)  3 (3.8%) | 26 (76.5%)  54 (71.1%) |
| 1. a.Using drops source of tension or conflict with my child  - Placebo - Atropine 0.01% | 0 (0.0%)  1 (1.3%) | 1 (3.0%)  3 (3.8%) | 30 (90.9%)  73 (90.0%) |
| b. Using drops source of tension or conflict with another family member   - Placebo - Atropine 0.01% | 0 (0.0%)  0 (0.0%) | 0 (0.0%)  1 (1.3%) | 32 (94.1%)  72 (90.0%) |
| c. Using drops source of tension or conflict with my child’s babysitter or teacher   - Placebo - Atropine 0.01% | 0 (0.0%)  0 (0.0%) | 0 (0.0%)  0 (0.0%) | 28 (84.9%)  51 (78.2%) |
| 1. Difficulty with near work  - Placebo - Atropine 0.01% | 0 (0.0%)  1 (1.3%) | 0 (0.0%)  1 (1.3%) | 32 (97.0%)  73 (94.8% |
| 1. Worry that child will become injured while on the drops  - Placebo - Atropine 0.01% | 0 (0.0%)  0 (0.0%) | 1 (3.0%)  1 (1.3%) | 30 (90.9%)  77 (96.3%) |
| 1. Child complains when it is time to put drops in  - Placebo - Atropine 0.01% | 0 (0.0%)  4 (5.0%) | 1 (3.0%)  10 (12.5%) | 29 (87.9%)  62 (77.5%) |
| 1. Child can see well while on the drops  - Placebo - Atropine 0.01% | 16 (50.0%)  36 (45.6%) | 10 (31.3%)  29 (36.7%) | 5 (15.6%)  12 (15.2%) |
| 1. Drops make child’s eye or eyelid red or irritated  - Placebo - Atropine 0.01% | 0 (0.0%)  1 (1.3%) | 3 (9.1%)  3 (3.8%) | 29 (87.9%)  70 (89.7%) |
| 1. Worry that child does not get drops often enough  - Placebo - Atropine 0.01% | 2 (6.3%)  4 (5.1%) | 4 (12.5%)  8 (10.1%) | 26 (81.3%)  64 (81.0%) |
| 1. Child more clumsy and uncoordinated while on the drops  - Placebo - Atropine 0.01% | 0 (0.0%)  0 (0.0%) | 0 (0.0%)  2 (2.5%) | 32 (97.0%)  74 (92.5%) |
| 1. Other children stare at my child when the drops are in*^†^* | - | - | - |
| 1. Believe that drops will improve child’s vision  - Placebo - Atropine 0.01% | 17 (51.5%)  43 (54.5%) | 14 (42.4%)  31 (39.2%) | 2 (6.1%)  5 (6.3%) |
| 1. Using the drops makes it difficult for my child to play with blocks or toys*^§^* | - | - | - |
| 1. Sometimes forget to put drops in  - Placebo - Atropine 0.01% | 10 (31.3%)  22 (27.5%) | 2 (6.3%)  7 (8.8%) | 13 (40.6%)  33 (41.3%) |
| 1. Worry that drops will make my child feel different from other children  - Placebo - Atropine 0.01% | 0 (0.0%)  1 (1.3%) | 0 (0.0%)  1 (1.3%) | 30 (96.8%)  73 (94.8%) |

*Some questions were not analysed as these were not relevant to the study as ^†^the eyedrops were instilled at night in the child’s home or ^§^the question is meant for children younger than the current cohort (6–16 years), rendering many “Not applicable” response to those questions.*
